# Supplementary material for: Systematic evaluation of high-level visual deficits and lesions in posterior cerebral artery stroke
Source: Brain Commun. 2023 Feb 28;5(2):fcad050. doi: 10.1093/braincomms/fcad050 (PMC10018645; doi:10.1093/braincomms/fcad050)
Supplement: fcad050_Supplementary_Data [file fcad050_supplementary_data.docx]

**Supplementary material for “Systematic evaluation of high level visual deficits and lesions in posterior cerebral artery stroke”**

Ro Julia Robotham^1,†^, Grace E Rice^2,†^, Alex P Leff^3^, Matthew A Lambon Ralph^2^ & Randi Starrfelt^1^*

**Supplementary Table 1. Patient recruitment breakdown ^1^**

|  | **Manchester** | **London** |
| --- | --- | --- |
| Identified (posterior cerebral artery stroke + provisional BoB criteria) | 109 | 91 |
| Not meeting inclusion criteria | 24 | 12 |
| Declined participation | 6 | 17 |
| Unable to contact | 51 | 15 |
| Completed testing but excluded from analysis | 4 | 3 |
| *Additional lesion outside posterior cerebral artery* | *2* | *0* |
| *Posterior cerebral artery without cortical involvement* | *2* | *1* |
| *Did not complete testing* | 0 | *2* |
| **Recruited & Completed Testing** | **23** | **41** |

**Supplementary Table 2. Comparison of demographics for laterality subgroups.**

| Demographics | Left | Bilateral | Right |
| --- | --- | --- | --- |
| N | 32 | 9 | 23 |
| Age | 63.9 (11.6) | 57.6 (10.7) | 57.9 (15.2) |
| Education (years) | 14.0 (2.5) | 13.8 (3.6) | 14.3 (2.6) |
| Time since stroke (months) | 42.3 (48.0) | 40.0 (28.5) | 42.0 (59.4) |
| **Comparison of demographics for laterality groups** | | | |
|  | Left vs Right  (*df* = 53) | Left vs Bilateral (*df* = 39) | Right vs Bilateral (*df* = 30) |
| Age | *t* = 1.69, *p* = 0.10 | *t* = 1.48, *p* = 0.15 | *t* = 0.06, *p* = 0.95 |
| Education (years) | *t* = 0.42, *p* = 0.68 | *t* = 0.18, *p* = 0.86 | *t* = 0.42, *p* = 0.68 |
| Time since stroke (months) | *t* = 0.02, *p* = 0.98 | *t* = 0.14, *p* = 0.89 | *t* = 0.09, *p* = 0.92 |

**Supplementary Table 3. Component loading tables for the unrotated PCA composite scores.** Any value above 0.5 is considered to be significant – all tests load significantly on their respective composite scores with the exception of the RT in the picture naming task (which falls just below)

**Word composite**

| **Test** | **Component Loading** |
| --- | --- |
| Word Reading (Accuracy) | **0.916** |
| Word Reading (3 letter words RT) | **0.908** |
| Lexical Decision (Real word RT) | **0.873** |
| Word Delayed Matching (Accuracy) | **0.870** |
| Word Surprise Recognition (RT) | **0.865** |
| Lexical Decision (Accuracy) | **0.858** |
| Word Delayed Matching (RT) | **0.814** |
| Word Surprise Recognition (Accuracy) | **0.514** |

**Object composite**

| **Test** | **Component Loading** |
| --- | --- |
| Object Decision (Accuracy) | **0.854** |
| Object Delayed Matching (RT) | **0.799** |
| Object Surprise Recognition (RT) | **0.793** |
| Picture Naming (Accuracy) | **0.775** |
| Object Delayed Matching (Accuracy) | **0.754** |
| Object Decision (Real Object RT) | **0.738** |
| Object Surprise Recognition (Accuracy) | **0.688** |
| Picture Naming (RT) | **0.401** |

**Face composite**

| **Test** | **Component Loading** |
| --- | --- |
| Famous Face Recognition (Accuracy) | **0.894** |
| Famous Face Naming (Accuracy) | **0.849** |
| Face Delayed Matching (Accuracy) | **0.841** |
| Face Surprise Recognition (Accuracy) | **0.822** |
| Face Familiarity (Accuracy) | **0.810** |
| Face Familiarity (Familiar RT) | **0.808** |
| Face Surprise Recognition (RT) | **0.806** |
| Face Delayed Matching (RT) | **0.718** |

**Supplementary Table 4:** **Lesion laterality, lesion volume, composite scores, and patterns of significant deficits (age as covariate; W = Words, O = Objects; F = Faces)**

| **Number** | **Participant** | **Laterality of lesion** | **Lesion volume** | **Age** | **WORDS** | **OBJECTS** | **FACES** | **Deficit** |
| --- | --- | --- | --- | --- | --- | --- | --- | --- |
| PL501 | Patient | L | 15,16 | 68 | 0,46 | 0,63 | -0,02 | None |
| PL502 | Patient | Bilat | 22,78 | 55 | 0,35 | 0 | -0,11 | OF |
| PL503 | Patient | L | 91,35 | 87 | -3,54 | -2,92 | -2,33 | WOF |
| PL504 | Patient | R | 53,80 | 85 | -0,07 | -0,74 | -0,07 | WO |
| PL505 | Patient | R | 126,14 | 69 | -1,15 | -1,44 | -2,66 | WOF |
| PL506 | Patient | L | 0,46 | 65 | 0,58 | 0,09 | 0,08 | None |
| PL507 | Patient | L | 15,94 | 70 | 0,01 | -1,13 | -1,07 | WOF |
| PL508 | Patient | L | 12,58 | 62 | -0,06 | 0,21 | 0,22 | W |
| PL510 | Patient | L | 12,17 | 67 | -0,06 | -0,51 | -0,65 | WOF |
| PL511 | Patient | L | 2,30 | 52 | 0,61 | 0,96 | 1,16 | None |
| PL513 | Patient | Bilat | 112,85 | 66 | -1,21 | -1,1 | -2,5 | WOF |
| PL514 | Patient | R | 27,49 | 71 | 0,05 | 0 | 0,58 | W |
| PL515 | Patient | L | 43,30 | 60 | -0,42 | 0,67 | 0,55 | W |
| PL516 | Patient | L | 53,78 | 65 | -0,35 | 0,14 | -0,63 | WF |
| PL517 | Patient | R | 15,06 | 62 | 0,58 | 0,22 | 0,7 | None |
| PL518 | Patient | Bilat | 51,82 | 52 | 0,4 | -0,07 | -1,47 | OF |
| PL519 | Patient | R | 18,65 | 52 | 0,17 | 0,5 | 0,6 | W |
| PL520 | Patient | R | 8,34 | 52 | -0,53 | -0,17 | -0,46 | WOF |
| PL521 | Patient | R | 62,51 | 62 | -0,12 | -0,53 | -2,23 | WOF |
| PL522 | Patient | R | 101,02 | 57 | 0 | -1,65 | -0,26 | WOF |
| PL523 | Patient | L | 13,85 | 57 | 0,5 | 0,28 | 0,83 | None |
| PL524 | Patient | L | 1,81 | 61 | 0,63 | 0,25 | 1,08 | None |
| PL525 | Patient | L | 58,66 | 80 | -0,7 | -0,46 | 0,17 | WO |
| PL526 | Patient | Bilat | 66,78 | 65 | -3,96 | -3,58 | -3,54 | WOF |
| PL527 | Patient | L | 28,32 | 76 | 0,04 | -0,01 | -0,37 | WF |
| PL528 | Patient | R | 53,79 | 84 | -0,18 | -1,44 | -1,02 | WOF |
| PL529 | Patient | L | 46,52 | 74 | -2,99 | -1,54 | -1,61 | WOF |
| PL530 | Patient | L | 40,52 | 64 | -0,28 | -0,22 | -0,21 | WOF |
| PL531 | Patient | L | 51,82 | 68 | -1,4 | -0,72 | -0,13 | WO |
| PL533 | Patient | R | 11,76 | 70 | 0,18 | 0,34 | 0,12 | W |
| PL534 | Patient | L | 26,23 | 75 | 0,36 | 0,44 | 0,09 | None |
| PL535 | Patient | R | 2,97 | 71 | 0,45 | -0,33 | -0,41 | OF |
| PL536 | Patient | R | 26,80 | 56 | 0,58 | 0,65 | 0,91 | None |
| PL537 | Patient | R | 4,17 | 27 | 0,13 | 0,33 | 0,31 | WO |
| PL538 | Patient | L | 48,64 | 83 | -1 | -1,93 | -2,56 | WOF |
| PL539 | Patient | R | 0,61 | 34 | 0,47 | 0,57 | 0,65 | None |
| PL540 | Patient | R | 1,49 | 28 | 0,61 | 0,83 | 0,73 | None |
| PL541 | Patient | Bilat | 76,74 | 34 | -0,47 | -1,38 | -1,74 | WOF |
| PL543 | Patient | Bilat | 18,10 | 52 | 0,29 | 0,62 | 0,04 | None |
| PL544 | Patient | R | 13,47 | 55 | -0,07 | -0,28 | 0,55 | WO |
| PL545 | Patient | Bilat | 57,42 | 62 | -0,38 | -0,94 | -0,53 | WOF |
| PM001 | Patient | L | 94,30 | 66 | -4,17 | -2,47 | -1,68 | WOF |
| PM002 | Patient | L | 90,27 | 51 | -1,28 | -1,49 | 0,45 | WO |
| PM004 | Patient | L | 103,63 | 58 | -2,78 | -0,24 | 0,11 | WO |
| PM006 | Patient | Bilat | 23,94 | 67 | 0,03 | -0,87 | -1,46 | WOF |
| PM007 | Patient | L | 46,28 | 63 | -1,53 | -1,19 | -2,31 | WOF |
| PM008 | Patient | L | 0,01 | 47 | 0,49 | 0,87 | 0,49 | None |
| PM009 | Patient | Bilat | 122,28 | 65 | -2,81 | -3,73 | -2,58 | WOF |
| PM010 | Patient | R | 4,14 | 52 | 0,5 | 0,84 | 0,72 | None |
| PM011 | Patient | L | 14,63 | 71 | -0,68 | -1,7 | -1,26 | WOF |
| PM012 | Patient | R | 1,55 | 46 | 0,44 | 0,83 | 0,92 | None |
| PM014 | Patient | L | 4,90 | 38 | 0,51 | 0,89 | 0,34 | None |
| PM015 | Patient | L | 15,18 | 44 | 0,17 | 0,68 | 0,71 | W |
| PM018 | Patient | L | 7,70 | 42 | -2,96 | -2,48 | -1,5 | WOF |
| PM019 | Patient | L | 8,00 | 70 | -0,19 | -0,46 | -0,14 | WO |
| PM021 | Patient | L | 21,63 | 74 | 0,42 | 0,32 | 0,31 | None |
| PM022 | Patient | L | 0,96 | 57 | 0,47 | 0,52 | 0,86 | None |
| PM023 | Patient | L | 19,82 | 71 | 0,58 | 0,44 | 0,84 | None |
| PM024 | Patient | R | 112,03 | 66 | -0,14 | -0,93 | -1,49 | WOF |
| PM025 | Patient | R | 62,70 | 70 | 0,19 | -0,16 | -0,43 | WF |
| PM026 | Patient | R | 81,42 | 62 | 0,16 | -1,27 | 0,04 | WO |
| PM028 | Patient | L | 25,77 | 60 | 0,33 | 0,28 | 0,09 | None |
| PM030 | Patient | R | 4,42 | 50 | 0,42 | 0,43 | 0,71 | None |
| PM031 | Patient | R | 4,15 | 51 | 0,67 | 1,16 | 1,01 | None |
| CL801 | Control | - | - | 57 | 0,17 | -0,06 | -0,04 | - |
| CL803 | Control | - | - | 62 | 0,47 | 0,93 | 0,4 | - |
| CL804 | Control | - | - | 38 | 0,57 | 0,94 | 0,97 | - |
| CL805 | Control | - | - | 56 | 0,23 | 0,09 | 0,67 | - |
| CL806 | Control | - | - | 55 | 0,36 | 0,6 | 0,86 | - |
| CL809 | Control | - | - | 34 | 0,35 | 0,49 | 0,22 | - |
| CL810 | Control | - | - | 60 | 0,63 | 0,89 | 1 | - |
| CL811 | Control | - | - | 35 | 0,69 | 0,96 | 0,68 | - |
| CL813 | Control | - | - | 79 | 0,46 | 0,55 | 0,43 | - |
| CL814 | Control | - | - | 30 | 0,57 | 0,9 | 0,95 | - |
| CL815 | Control | - | - | 73 | 0,43 | 0,41 | -0,2 | - |
| CL816 | Control | - | - | 48 | 0,42 | 0,53 | 0,75 | - |
| CL817 | Control | - | - | 30 | 0,68 | 0,65 | 0,57 | - |
| CL818 | Control | - | - | 50 | 0,41 | 0,74 | 0,76 | - |
| CM301 | Control | - | - | 70 | 0,58 | 0,31 | 0,28 | - |
| CM303 | Control | - | - | 42 | 0,6 | 1,02 | 1,03 | - |
| CM304 | Control | - | - | 57 | 0,7 | 0,84 | 0,34 | - |
| CM305 | Control | - | - | 54 | 0,59 | 1,04 | 1,06 | - |
| CM306 | Control | - | - | 45 | 0,7 | 1,19 | 0,94 | - |
| CM307 | Control | - | - | 70 | 0,56 | 0,5 | 0,67 | - |
| CM308 | Control | - | - | 71 | 0,58 | 0,97 | 0,61 | - |
| CM309 | Control | - | - | 62 | 0,51 | 0,73 | 0,98 | - |
| CM310 | Control | - | - | 72 | 0,44 | 0,58 | 0,48 | - |
| CM311 | Control | - | - | 80 | 0,57 | 0,08 | -0,08 | - |
| CM312 | Control | - | - | 72 | 0,41 | -0,26 | 0,46 | - |
| CM313 | Control | - | - | 69 | 0,53 | 0,11 | 0,23 | - |
| CM314 | Control | - | - | 26 | 0,6 | 1,26 | 0,66 | - |
| CM315 | Control | - | - | 67 | 0,58 | 0,77 | 0,35 | - |
| CM316 | Control | - | - | 70 | 0,4 | 0,31 | 0,12 | - |
| CM317 | Control | - | - | 67 | 0,66 | 0,58 | 0,86 | - |
| CM318 | Control | - | - | 67 | 0,54 | 0,11 | -0,79 | - |
| CM319 | Control | - | - | 76 | 0,23 | -0,15 | -0,06 | - |
| CM320 | Control | - | - | 66 | 0,35 | 0,43 | -0,25 | - |
| CM321 | Control | - | - | 84 | 0,17 | -0,44 | 0,08 | - |
| CM322 | Control | - | - | 69 | 0,46 | 0,58 | 0,6 | - |
| CM323 | Control | - | - | 68 | 0,42 | 0,1 | 0,45 | - |
| CM325 | Control | - | - | 68 | 0,38 | 0,94 | 0,01 | - |
| CM326 | Control | - | - | 62 | 0,51 | 0,67 | 0,26 | - |
| CM327 | Control | - | - | 75 | 0,2 | -0,31 | -0,19 | - |
| CM328 | Control | - | - | 78 | 0,45 | 0,4 | 0,38 | - |
| CM329 | Control | - | - | 64 | 0,75 | 0,69 | 0,74 | - |
| CM330 | Control | - | - | 71 | 0,6 | 0,6 | 0,74 | - |
| CM331 | Control | - | - | 73 | 0,62 | 0,97 | 0,92 | - |
| CM332 | Control | - | - | 67 | 0,52 | 0,75 | 0,89 | - |
| CM333 | Control | - | - | 71 | 0,48 | 0,35 | 0,84 | - |
| CM334 | Control | - | - | 70 | 0,54 | 0,7 | 0,8 | - |

**Supplementary Table 5: whole brain multiple regression analysis on the constituent PCA ROIs**

|  |  | Words (beta) | Objects (beta) | Faces (beta) |
| --- | --- | --- | --- | --- |
| Model 1 | L ILF | -0.62 *** |  |  |
| Model 2 | L ILF | -0.50 *** |  |  |
|  | L Occipital | -0.38 *** |  |  |
| Model 3 | L ILF | -0.49 *** |  |  |
|  | L Occipital | -0.39 *** |  |  |
|  | R pITG | -0.22 ** |  |  |
| Model 1 | Total Lesion Volume |  | -0.63 *** |  |
| Model 2 | Total Lesion Volume |  | -0.57 *** |  |
|  | L ILF |  | -0.23 * |  |
| Model 1 | Total Lesion Volume |  |  | -0.57 *** |
| Model 2 | Total Lesion Volume |  |  | -0.52 *** |
|  | R aITG |  |  | -0.23 * |
| Model 3 | Total Lesion Volume |  |  | -0.43 *** |
|  | R aITG |  |  | -0.22 * |
|  | Unilateral vs. Bilateral stroke |  |  | -0.22 * |
| Model 4 | Total Lesion Volume |  |  | -0.61 *** |
|  | R aITG |  |  | -0.21 * |
|  | Unilateral vs. Bilateral stroke |  |  | -0.27 ** |
|  | R Lingual gyrus |  |  | 0.29 * |

*p<0.05, **p<0.01, ***p<0.001

**Supplementary Material: Behavioral assessment details**

#### Delayed Matching and Surprise Recognition test of Words, Objects and Faces (WOF test)

Reason for inclusion: This test was included in order to compare recognition abilities across the categories of faces, words and objects. The experiment uses the same paradigm to assess face, word and object recognition, leading to easier comparison across categories.

About the test: The Delayed Matching Test and Surprise Recognition Test was developed specifically for the BoB-project and involves two parts: a delayed matching test and a surprise recognition test ^2^. The Delayed Matching Test assesses the ability to build a short-term representation of a stimulus and then match it with the same or a novel stimulus. The Surprise Recognition test that is administered directly after is an old/new recognition paradigm that assesses whether participants can recognize stimuli that were used in the Delayed Matching part of the test. They are not told that there will be a subsequent recognition test when performing the Delayed matching part. Processing of words, objects and faces are assessed independently in each part. With its two separate parts, a distinction can be made between recognition problems that are caused by a deficit in storing a representation over longer time from deficits related to problems in creating a short-term representation of a stimulus and matching it with a currently viewed stimulus.

Delayed Matching Test

Materials: For each category, four groups of three visually similar stimuli are used (12 uncropped faces, 12 words, and 12 objects; Figure 4). All images are in black and white. The faces were selected from the Radboud Faces Database^3^. All faces are presented in frontal view with neutral emotional expressions. Two clusters of three male faces and two clusters of three female faces are used. The three faces in a cluster have similar hairstyles and similar visual features (see Figure 4). For the word stimuli, four clusters of three 4-letter words are used. Words in the same group only differ by one letter. In group 1, the first letter changes, in group 2, the second letter changes; in group 3, the third letter changes; and in group 4, the fourth letter changes. The task can therefore not be performed by focusing on a single letter position. Words are presented in lowercase writing in Arial font. The object stimuli includes four clusters of images representing four different object categories: cars, butterflies, boots, and flowers.


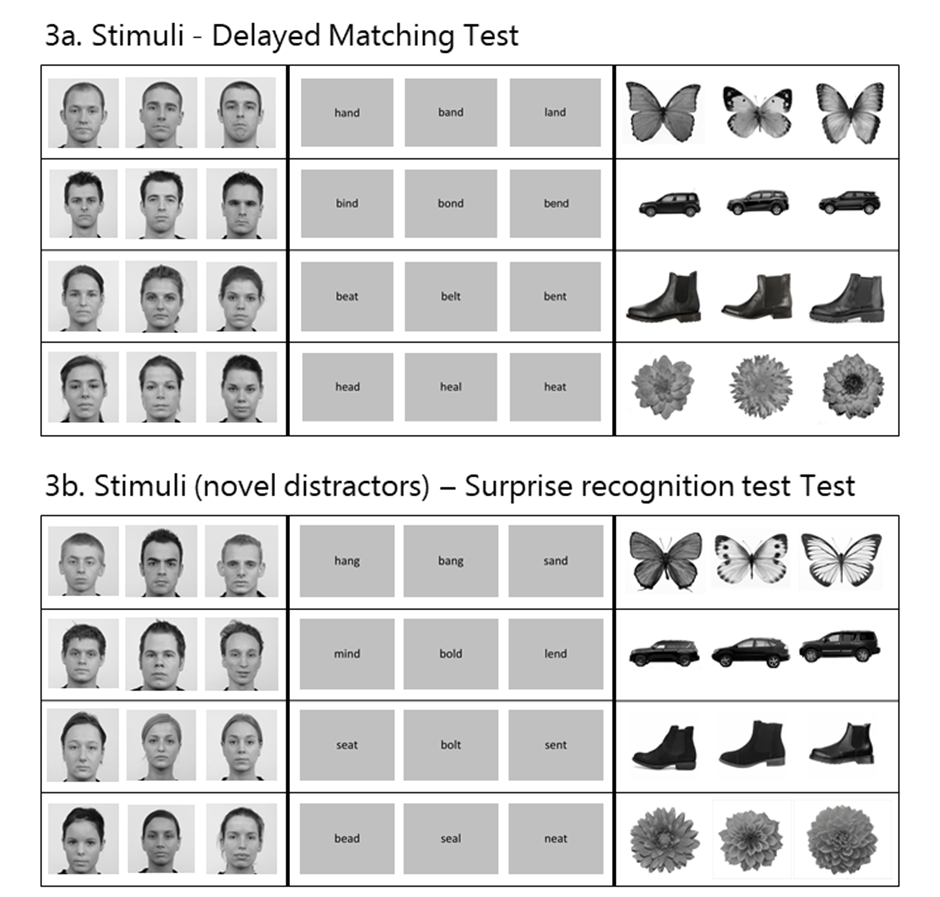


Figure 4. Stimuli – Delayed matching test

Procedure: The three categories are assessed in separate blocks in the following order: faces, words and objects. A practice session with four practice trials precedes each block. One trial consists of a target stimulus followed by a test stimulus (Figure 5). In 50% of trials, the test stimulus is the same as the target stimulus, and in 50% of trials, the test stimulus is a different stimulus (coming from the same cluster). Participants must determine via button-press whether the test and target stimuli is the same or different. Accuracy and reaction times are recorded. To avoid the task being a change detection task, test images are presented in smaller dimensions (2/3) than the target images in 50% of trials and in larger dimensions (4/3) than the target images in 50% of trials. Each block (category) involves 48 trials and each cluster of three stimuli is assessed through 12 trials. Trials are presented randomly within a block.


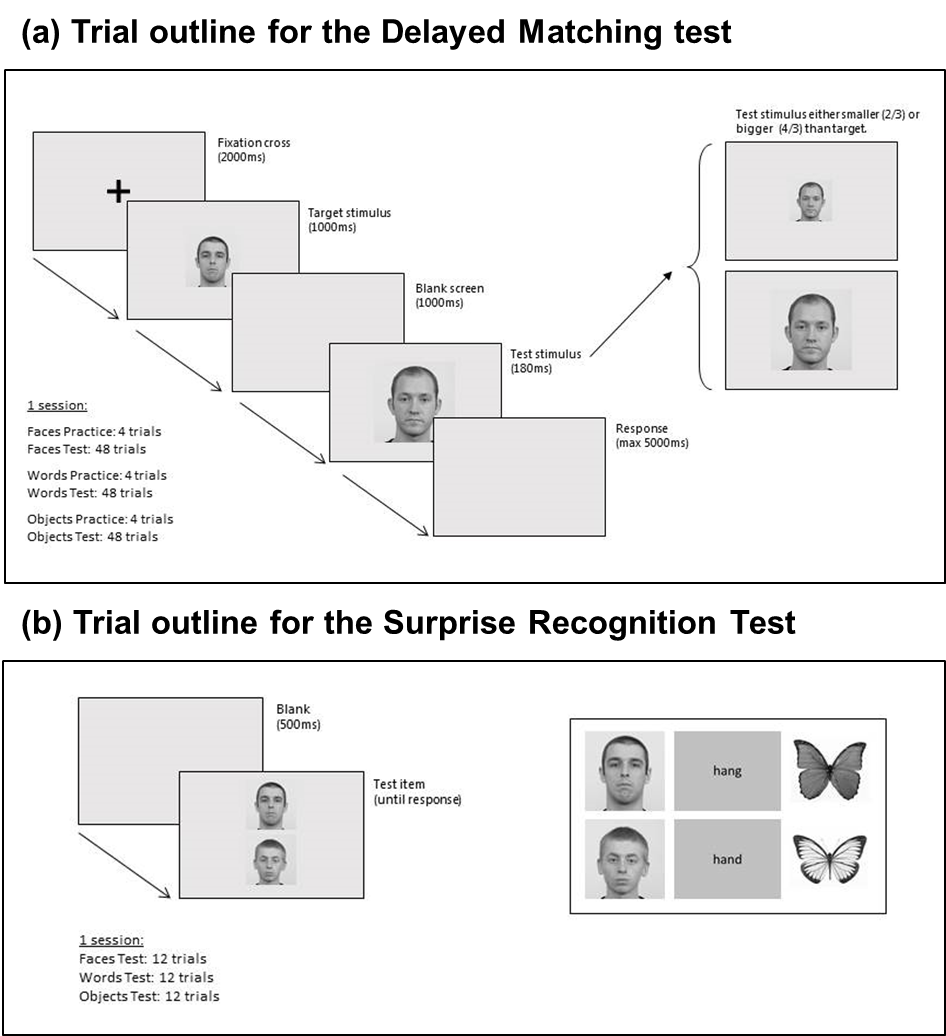


Figure 5: Trial outline for Delayed matching test

Surprise Recognition Test

Materials: The 36 stimuli used in the *Delayed Matching* part of the test are re-used in this part of the test (Figure 4), 12 novel faces, 12 novel words, and 12 novel objects are also included (Figure 6). The novel stimuli were selected so that they pairwise closely matched the stimuli used in the *Delayed Matching* part of the test. Each new face was selected to look highly similar to a face used in the *Delayed Matching* part of the test. Each new word differed from the words previously used with one letter only. Each new object was selected to look highly similar to one of the objects previously used. Similarity between images was not formally controlled.


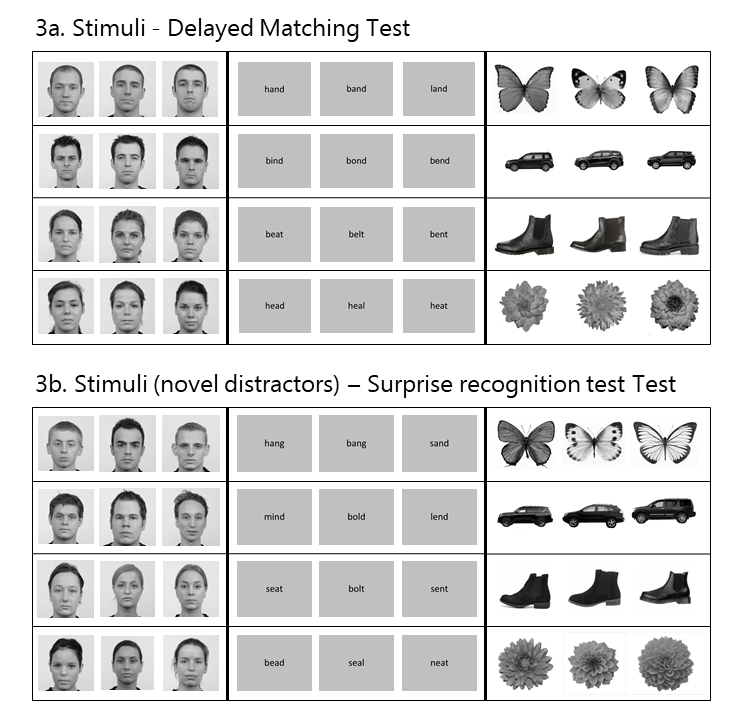


Figure 6. Stimuli – Novel distractors for Surprise recognition test

Procedure: The *Surprise Recognition* paradigm is run following a short break after the *Delayed Matching* paradigm. Categories are again presented in separate blocks and are presented in the same order as in the *Delayed Matching* paradigm: faces, words and objects. One trial consisted of a novel face and a target face being presented vertically on a screen. In 50% of trials the target is on top and 50% of trials the target is at the bottom of the screen. Participants are asked to determine which of the images they have seen before by pressing the ↑key or the ↓key. A trial ends when the participant presses a response key (Figure 7). Accuracy and reaction times are recorded. Each target face is presented once. There are therefore 12 trials in each block.


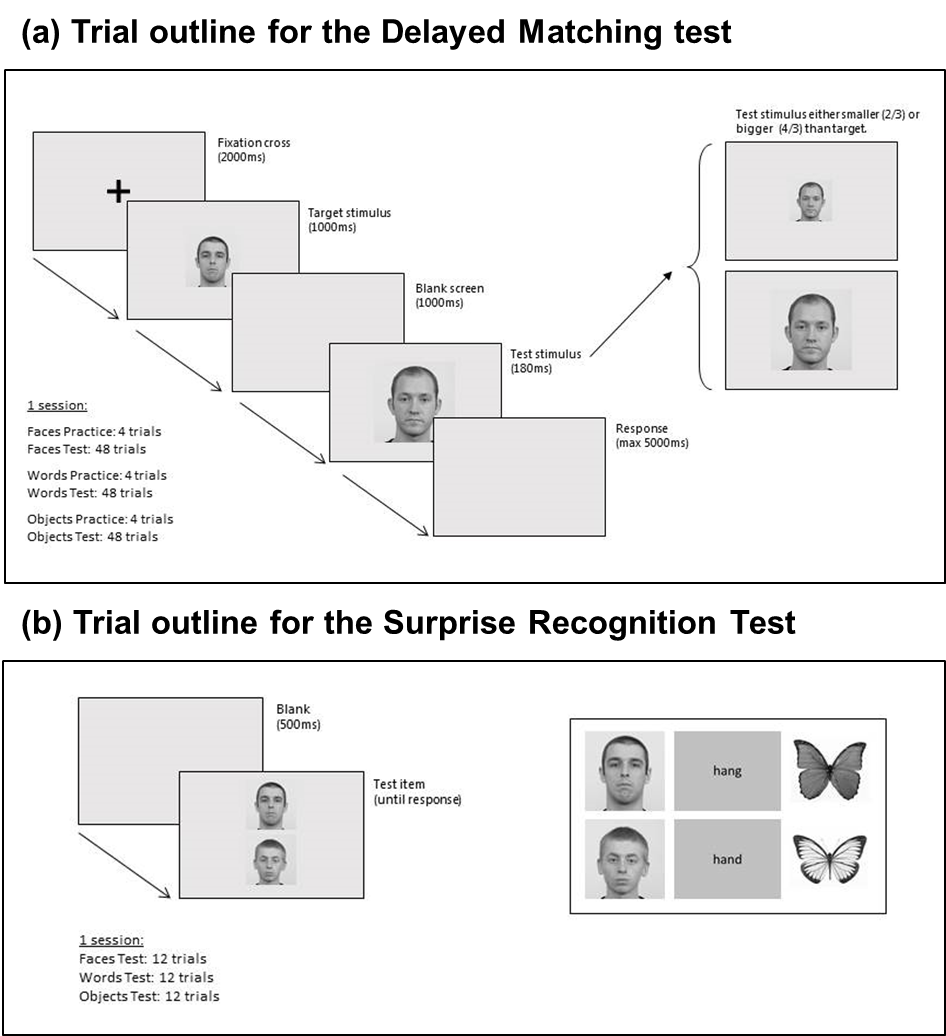


Figure 7: Trial outline for the Surprise recognition test

#### Lexical Decision / Object Decision / Face Familiarity Decision

Reason for inclusion: A task that involves determining whether an item is familiar (deciding whether one has seen a given stimulus before or not) was included for each category of interest: words (lexical decision: word or non-word), objects (object decision: object or non-object), and faces (face familiarity: famous or non-famous face). This enables the comparison of visual recognition abilities across categories, without the need for a verbal (naming) output. The lexical decision test involves deciding whether a letter-string stimulus is a word or a non-word, and is commonly used to assess reading abilities in acquired reading disorders ^4,5^ . Lexical decisions may be performed based on pre-semantic, visual lexical representations. The Object Decision test involves determining whether an image is depicting an object or a non-object. The nonobjects in the current project were chimeric nonobjects created from parts of real objects, and the test is considered to primarily reflect pre-semantic processing (visual long term memory).^6^ The Famous Face Familiarity Decision test assesses the ability to recognise a face as familiar. Participants must match the perceived face to a representation stored in long term memory. Participants are shown one face at a time and must determine if it is a famous face or a novel face. This task is a measure of both perceptual and semantic processing.

Lexical decision - About the test: A 60-item lexical decision task (30 words and 30 pseudowords) was administered to assess word recognition. Participants were presented with one stimulus at a time centrally on the screen and had to indicate via button-press as quickly and accurately as possible whether the letter-string was a word or not. Items were either 3, 5, or 7 letters in length. Non-words were phonologically plausible letter combinations. The main dependent variables were accuracy and correct response time. Stimuli were selected from the task used by Behrmann & Plaut (2014)^5^, and included half the stimuli from the original test. See supplementary material for list of words included in the task.

Object decision - About the test: The 72-item test that was included in the BoB-protocol has been described in many publications ^6,7^. The stimuli were presented one at a time centrally on a screen and participants were required to respond, via button-press, as quickly and accurately as possible whether the stimulus depicted a real object or a (chimeric) nonobject. The main dependent variables were accuracy and correct response time.

Face familiarity decision - About the test: This test contained 80 items, including the 40 famous faces included in the Famous Face Naming task. Faces were presented one at a time centrally on a screen and participants had to determine as quickly and accurately as possible via button-press whether the face was famous or not. The dependent measures were accuracy and correct response time. The test was designed specifically for the Back of the Brain project.

#### Word reading (length)

Reason for inclusion: This test measures response time and accuracy when reading words of different lengths and enables calculation of the word length effect, which is a core characteristic of pure alexia. Subjects with hemianopia typically also show a word length effect (although more modest).

About the test: The test has been used in previous investigations of pure alexia ^8,9^. Participants were asked to read 75 regularly spelled single words out-loud as quickly and accurately as possible. Items were either 3, 5, or 7 letters in length (25 words per length). Each item was displayed on the screen until a response was recorded or a maximum of 4 seconds. Correct response times from stimulus onset to vocal response were measured using a voice key. Accuracy was recorded by the experimenter and responses were recorded using a Dictaphone for the purposes of error analysis. Responses provided after more than four seconds were counted as errors. See supplementary material for list of words included in the test.

#### Picture Naming

Reason for inclusion: A picture naming test that has been used in previous studies ^10^ was included in the protocol to enable comparison of identification abilities across categories. Picture naming abilities can be compared to famous face naming abilities and word reading abilities.

About the test: Participants are required to name 45 black and white line drawings of objects as quickly and accurately as possible. The stimuli consists of 30 living items (animals, insects) and 15 non-living items (musical instruments, vehicles, tools). Within the living items there is a manipulation of “homomorphy” ^11^; the amount to which an items contour is shared with other exemplars within that category (15 living items had high homomorphy and 15 living items had low homomorphy). Previous studies have shown that posterior stroke patients may show category effects during naming, where performance is worse when naming living items compared to non-living items. The manipulation of homomorphy is included to test the hypothesis that any such category effects are due to low-level perceptual effects caused by the high homomorphy overlap in living items compared to non-living items (which tend to be more unique in their contour). The same voice key procedure described for the word reading tests was adopted for the BoB project. Items were presented on the screen until a response was made or for a maximum of 6 seconds. Accuracy was recorded by the experimenter.

#### Famous Face Naming

Reason for inclusion: A Famous Face Naming test was included to enable comparison of identification abilities across categories. Famous Face Naming abilities can be compared to Picture Naming Abilities and Word Reading abilities.

About the test: This test was used in a previous case-series investigation of posterior cerebral lesions^12^. The test contains 40 items, pictures of famous faces are presented one at a time centrally on a screen and participants are asked to name the person out loud as quickly and as accurately as possible. If they are unable to provide their name, recognition of the person is tested (e.g., provision of why the person is famous, what they do, where they live etc.). The main measure for this test was accuracy, reaction time data was not scored due to the extensive verbal output. Responses were scored according to whether the correct name was provided and whether correct semantic information was provided. The items included in this test were also included in the Face Familiarity test (the two tests were administered on different days, the face familiarity test first).

**References**

1. Rice GE, Kerry SJ, Robotham RJ, Leff AP, Ralph MAL, Starrfelt R. Category-selective deficits are the exception and not the rule: Evidence from a case-series of 64 patients with ventral occipito-temporal cortex damage. *Cortex,*. 2021;138:266-281.

2. Robotham RJ. *The Neuropsychology of Stroke in the Back of the Brain: Clinical and Cognitive Aspects*. 2019.

3. Langner O, Dotsch R, Bijlstra G, Wigboldus DHJ, Hawk ST, van Knippenberg A. Presentation and validation of the Radboud Faces Database. *Cognition and Emotion*. 2010;24(8):1377-1388. doi:10.1080/02699930903485076

4. Behrmann M, Plaut DC, Nelson J. A literature review and new data supporting an interactive account of letter-by-letter reading. *Cogn Neuropsychol*. 1998;15(1/2):7-51. doi:10.1080/026432998381212

5. Behrmann M, Plaut DC. Bilateral Hemispheric Processing of Words and Faces: Evidence from Word Impairments in Prosopagnosia and Face Impairments in Pure Alexia. *Cerebral Cortex*. 2014;24(4):1102-1118. doi:10.1093/cercor/bhs390

6. Gerlach C. Category-specificity in visual object recognition. *Cognition*. 2009;111(3):281-301. doi:<https://doi.org/10.1016/j.cognition.2009.02.005>

7. Starrfelt R, Habekost T, Gerlach C. Visual processing in pure alexia: A case study. *Cortex: A Journal Devoted to the Study of the Nervous System and Behavior*. 2010;46(2):242-255.

8. Habekost T, Petersen A, Behrmann M, Starrfelt R. From word superiority to word inferiority: visual processing of letters and words in pure alexia. *Cogn Neuropsychol*. 2014;31(5-6):413-436. doi:10.1080/02643294.2014.906398

9. Starrfelt R, Habekost T, Leff AP. Too little, too late: Reduced visual span and speed characterize pure alexia. *Cerebral Cortex*. 2009;19(12):2880-2890.

10. Roberts DJ, Woollams AM, Kim E, Beeson PM, Rapcsak SZ, Lambon Ralph MA. Efficient Visual Object and Word Recognition Relies on High Spatial Frequency Coding in the Left Posterior Fusiform Gyrus: Evidence from a Case-Series of Patients with Ventral Occipito-Temporal Cortex Damage. *Cerebral Cortex*. 2013;23(11):2568-2580. doi:10.1093/cercor/bhs224

11. Tranel D, Logan CG, Frank RJ, Damasio AR. Explaining category-related effects in the retrieval of conceptual and lexical knowledge for concrete entities : operationalization and analysis of factors. 1997;35(10)

12. Roberts DJ, Lambon Ralph MA, Kim E, et al. Processing deficits for familiar and novel faces in patients with left posterior fusiform lesions. *Cortex*. 2015;72(SI):79-96. doi:<http://dx.doi.org/10.1016/j.cortex.2015.02.003>
